# First record of supernumerary (B) chromosomes in electric fish (Gymnotiformes) and the karyotype structure of three species of the same order from the upper Paraná River basin

**DOI:** 10.3897/CompCytogen.v6i1.1752

**Published:** 2012-01-24

**Authors:** Valéria Pereira Mendes, Ana Luiza de Brito Portela-Castro, Horácio Ferreira Júlio-Júnior

**Affiliations:** 1 Universidade Estadual de Maringá, Departamento de Biologia Celular e Genetica, Avenida Colombo 5790, 87020-900 Maringá, PR, Brazil; 2 Universidade Estadual de Maringá, Departamento de Biologia Celular e Genetica, Núcleo de Pesquisas em Limnologia, Ictiologia e Aqüicultura (Nupélia, UEM), Avenida Colombo 5790, 87020-900 Maringá, PR, Brazil

**Keywords:** fish cytogenetics, B chromosomes, C-banding, ribosomal DNA, sex chromosomes

## Abstract

Cytogenetic studies were performed on the species *Apteronotus* prope *albifrons* Linnaeus, 1766, *Rhamphichthys hahni* Meinken, 1937 and *Brachyhypopomus gauderio* Giora & Malabarba, 2009, collected in the upper Paraná River floodplain, Porto Rico (PR), Brazil. *Apteronotus* prope *albifrons* showed a diploid number of 2n=24 chromosomes for both sexes and a karyotype formula of 14m+2sm+2st+6a (FN=42). Besides the standard karyotype, three specimens had one to three extra microchromosomes with inter- and intra-individual variations, which suggested the occurrence of B chromosomes in the species. The chromosomal data of *Rhamphichthys hahni*, described here for the first time, consists of 50 chromosomes and a formula comprised of 20m+24sm+6a (FN=94). *Brachyhypopomus gauderio* specimens demonstrated 2n=42 chromosomes infemales, all acrocentric, and 2n=41 chromosomes in males, with 40 acrocentric and 1 medium-sized metacentric chromosome. These differences concern with a multiple system of sexchromosome determination X_1_X_1_X_2_X_2_/X_1_X_2_Y (FN=42) in *Brachyhypopomus gauderio*. The analysis of nucleolar organizer regions by Ag-NOR and FISH 18S banding revealed a simple NOR system in *Apteronotus* prope *albifrons* and *Rhamphichthys hahni* and a multiple NOR system in *Brachyhypopomus gauderio*, that is unusual for Gymnotiformes fishes. Constitutive heterochromatin was mainly found in the pericentromere region in most of the chromosomes of the three species, although each species had its own peculiarities. The B chromosomes in *Apteronotus* prope *albifrons* demonstrated heterochromatin positioned in the centromeric and telomeric regions whereas *Rhamphichthys hahni* presented conspicuous blocks of heterochromatin on the long arms in three submetacentric pairs. *Brachyhypopomus gauderio* showed blocks of heterochromatin on the long arm in the interstitial and telomere positions. The finding of B chromosomes in *Apteronotus* prope *albifrons* represents the first description of these elements in the Gymnotiformes order. Although the karyotype of this species is similar with that described for populations in the Amazon basin, the presence of B chromosomes could represent a specific characteristic of this population. A comparative analysis of karyotypes of *Rhamphichthys hahni* with other species of the genus showed a relatively conservative structure suggesting 2n=50 as a common number in this group. The karyotype of *Brachyhypopomus gauderio*, a new species, provides an important reference for future chromosome studies of the *Brachyhypopmus* Mago-Lecia, 1994, and it might be also significant for cytotaxonomy in this group. The cytogenetic data also demonstrate the need of more comparative cytogenetic studies in the families of the highly diversified and taxonomically difficult complex Gymnotiformes.

## Introduction

The order Gymnotiformes is a group with high species diversity and about 179 species have been listed so far (Crampton 2011).

Karyotype diversity is well known in Gymnotiformes, especially in the genera *Gymnotus* Linnaeus, 1758 and *Eigenmannia* Jordan et Evermann, 1896. Regarding this order, the diploid number ranges from 2n = 22 or 24 in *Apteronotus albifrons* Linnaeus, 1766 (Hinegardner and Rosen 1972, Almeida-Toledo et al. 1981, respectively) to 2n=54, the highest diploid number recorded in *Gymnotus carapo* Linnaeus, 1758 and *Gymnotus mamiraua* Albert et Crampton, 2001 (Foresti et al. 1984, Milhomem et al. 2007, respectively).

The karyotype variability in Gymnotiformes also involves simple and multiple sex chromosome systems such as those registered in Gymnotidae, Hypopomidae and Sternopygidae species. The sex chromosome systems XX/XY and ZZ/ZW were described in *Eigenmannia virescens* Valenciennes, 1836 (Almeida-Toledo et al. 2001, Silva et al. 2009) and X_1_X_1_X_2_X_2_/X_1_X_2_Y in *Eigenmannia* sp, (Almeida-Toledo et al. 2000a), *Gymnotus* sp. (Silva and Margarido 2005) and *Brachyhypopomus gauderio* (earlier misidentified as *Brachyhypopomus pinnicaudatus* Hopkins, 1991 (Almeida-Toledo et al. 2000b). Cytogenetic information on the Apteronotidae, Hypopomidae and Rhamphichthyidae families is very scarce in the literature. Table 1 shows the cytogenetic data reported for these families to date.

**Table 1.** Cytogenetic data available for Apteronotidae, Rhamphichthyidae and Hypopomidae families. FN: fundamental number; m: metacentric; sm: submetacentric; st: subtelocentric; a: acrocentric; AM: Amazonas; PR: Paraná; SP: São Paulo; PA:Pará.

**Family/species**	**Locality**	**2n**	**FN**	**Karyotype**	**Sex chromo-somes**	**Reference**
**Apteronotidae**						
*Apteronotus albifrons*	Amazon	24	42	14m+2sm+2st+6a		Howell (1972)
*Apteronotus albifrons*	Marajó Island, PA	24	42	12m+4sm+2st+6a		Almeida-Toledo et al. (1981)
*Apteronotus* prope *albifrons*	Upper PR, PR	24	42	14m+2sm+2st+6a		Present study
*Apteronotus albifrons*		22				Hinegardner and Rosen (1972)
*Apteronotus* sp.	São Paulo	52	98	46m/sm+ 6st/a		Almeida-Toledo et al. (2007)
*Parapteronotus bonaparti* (*Apteronotus anas*)	Manaus (AM)	52	94	30m+12sm+10a		Almeida-Toledo et al. (2007)
*Parapteronotus hasemani* (*Apteronotus*)	Manaus, AM	52	94	26m+16sm+10a		Almeida-Toledo et al. (2007)
**Rhamphichthyidae**						
*Rhamphichthys hani*	Upper PR, PR	50	94	20m+24sm+6a		Present study
*Rhamphichthys* prope *pantherinus*	Amazon	52	100	38m+10sm+4st		Almeida-Toledo (1978)
*Rhamphichthys marmoratus*	Amazon	50	94	44m/sm+6st/a		Silva (2010)
*Rhamphichthys rostratus*	Amazon	50	92	42m/sm+8a		Silva (2010)
**Hypopomidae**						
*Brachyhypopomus brevirostris*	Amazon	36	42	4m+2sm+8st+22a		Almeida-Toledo (1978)
*Brachyhypopomus gauderio*	Upper PR, PR	♀ 42	42	42a	X_1_X_1_X_2_X_2_	Present study
		♂ 41	42	1m+40a	X_1_X_2_Y	
	Tietê River, SP	♀ 42	42	42a	X_1_X_1_X_2_X_2_	Almeida-Toledo et al. (2000b)
		♂ 41	42	1m+40a	X_1_X_2_Y	(as *Brachyhypopomus pinnicaudatus*)
*Hypopomus artedi*	Amazon	38	70	32m/sm+6st/a		Almeida-Toledo (1978)
*Hypopygus lepturus*	Amazon	50	96	16m+20sm+10st+4a		Almeida-Toledo (1978)

In spite of extensive karyotype variability, a review of the cytogenetic studies of Neotropical freshwater fish shows that, to date, there is no record of B chromosomes (or supernumerary chromosomes) in Gymnotiformes (Carvalho et al. 2008, Oliveira et al. 2009). These chromosomes are not essential to cell functioning and may be derived from autosomes and sex chromosomes in intra- and interspecies crosses (Camacho et al. 2000). The B chromosomes are well documented in fishes and have been reported in more than 60 species. Characiformes appear to have a high proportion of species with B chromosomes: 50.8% of a total of 61 species examined (Carvalho et al. 2008). Other groups show a lower prevalence of species with B chromosomes; for example, 34.42% in Siluriformes and 8.19% in Perciformes, and other orders, such as Beloniformes, Cyprinodontiformes and Synbranchiformes, have only one species with B chromosomes (1.63% per order) (Carvalho et al. 2008).

Although Neotropical fishes show considerable variability regarding the number of B chromosomes (1–16), usually 1–4 chromosomes are present. The B chromosomes show wide variations in size, from very small (micro), as in *Moenkhausia sanctaefilomenae* Steindachner, 1907 (Foresti et al. 1989, Portela-Castro et al. 2001) and *Rineloricaria pentamaculata* Langeani et Araujo, 1994 (Errero-Porto 2010), small, asin *Cyphocharax modestus* Fernández-Yépez, 1948 (Vênere et al. 1999), medium sized, as in *Rhamdia quelen* Quoy et Gaimard, 1824 and *Rhamdia branneri* Haseman, 1911 (Fenocchio and Bertollo 1990, Abucarma and Martins-Santos 2001, respectively) and large, such as the different species of the genus *Astyanax* Baird et Girard, 1854 (Moreira-Filho et al. 2004).

Methodologies such as C-banding have revealed the nature of heterochromatic B chromosomes with repetitive DNA sequences (Camacho et al. 2000). Totally heterochromatic B chromosomes constitute a common situation in many species of Neotropical fishes; however, they can be completely euchromatic, as in *Moenkausia sanctefilomenae* (Foresti et al. 1989), *Steindachnerina insculpta* Fernández-Yépez, 1948 (Oliveira and Foresti 1993), *Characidium* prope *zebra* (Vênere et al. 1999) and *Rhamdia quelen* (Moraes et al. 2009), or partially heterochromatic, as in some populations of “*Astyanax scabripinnis* complex” (Moreira-Filho et al. 2004), *Rhamdia hilarii* Valenciennes, 1840 (Fenocchio and Bertollo 1990) and *Rhamdia quelen* (Moraes et al. 2009).

## Material and methods

**Figure 1.**
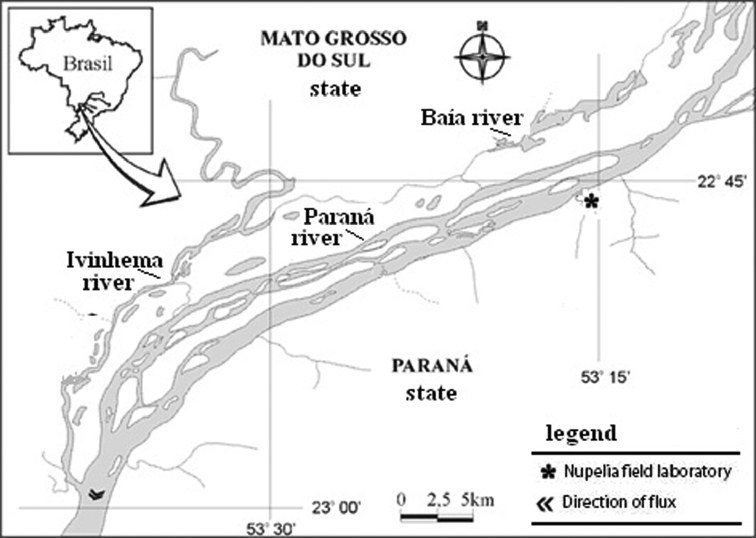
Collections sites of studied species**.** Map of Brazil showing the localization of Paraná river, in the Paraná state. Hhydrographic map of the floodplain of the Upper Paraná River and its tributaries, Baía and Ivinhema Rivers.

**Figure 2 a–c.**
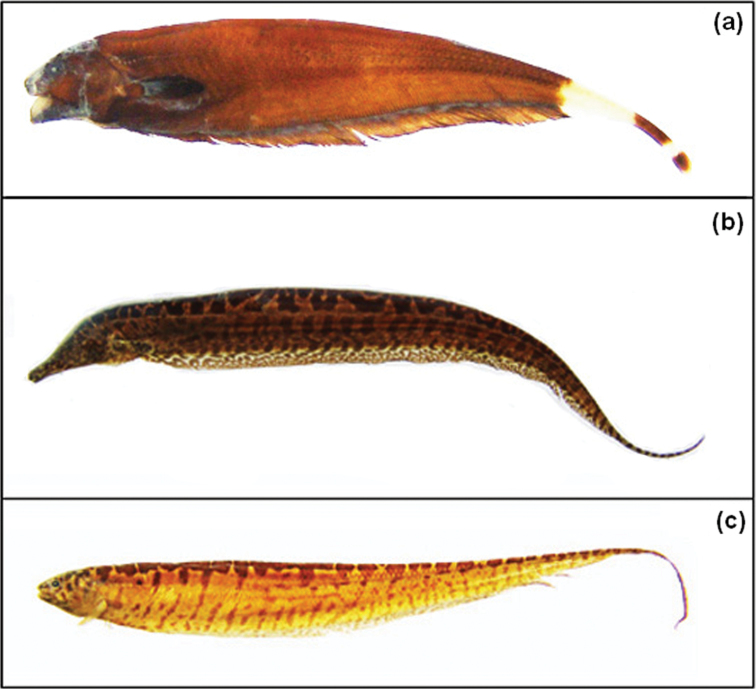
Specimens of: **a** *Apteronotus* prope *albifrons* (102,7 mm SL, NUP9621) **b** *Rhamphichthys hahni* (244,4 mm SL, NUP9623) and **c** *Brachyhypopomus gauderio* (140,9mm SL, NUP9622).

We analysed 51 specimens (24 males and 27 females) of *Brachyhypopomus gauderio*, 6 specimens of *Apteronotus* prope *albifrons* (4 males and 2 females) and 19 specimens of *Rhamphichthys hahnii* (8 males, 7 females and 4 undetermined sex). The specimens were collected in rivers (Baía and Ivinhema) and lagoons of the upper Paraná River floodplain near the town of Porto Rico (PR), Brazil (Fig. 1). Voucher specimens were deposited in the fish collection of the Research Nucleus in Limnology, Ichthyology and Aquaculture (Nupélia), Universidade Estadual de Maringá, PR Brazil, as *Apteronotus* prope *albifrons* (NUP9621), *Rhamphichthys hahni* (NUP9623) and *Brachyhypopomus gauderio*, (earlier misidentified as *Brachyhypopomus* prope *pinnicaudatus*, NUP9622). *Brachyhypopomus gauderio* represents a new species of the southern Brazil, Uruguay and Paraguay described by Giora and Malabarba (2009). Fig. 2 shows photographs of each species.

### Conventional staining

We obtained metaphase chromosomes from kidney cells using the air-drying technique proposed by Bertollo et al. (1978) and stained with 5% Giemsa in phosphate buffer (pH 6.8). We used benzocaine solution to anaesthetize the fish before sacrificing them. We used the arm ratio criteria (AR) to characterize chromosome morphology, as suggested by Levan et al. (1964): metacentric (m), submetacentric (sm), subtelocentric (st), and acrocentric (a) chromosomes.

### Chromosome banding

We used the C-banding method and staining with Giemsa after treatments with 0.1M HCl, Ba(OH)_2 _and 2×SSC solutions to analyse the distribution of heterochromatin, as described by Sumner (1972), and we used thesilver nitrate staining method (Ag-NOR) to identify the nucleolus organizer regions (NORs), as described by Howell and Black (1980).

### Fluorescent in-situ hybridization (FISH)

The probes used to detect 18S rDNA in the FISH analyses were obtained by amplified and cloned fragments of *Oreochromis niloticus* Linnaeus, 1758 (kindly provided by Dr Cesar Martins of the Universidade Estadual Paulista, Botucatu SP Brazil). We used the methods of Heslop-Harrison et al. (1991) and Cuadrado and Jouve (1994) to perform the FISH analyses, with modifications by Swarça et al. (2001). The probes were labelled with biotin 14-dATP via nick translation (Bio Nick Labeling System - Gibco, BRL). We incubated the slides with RNase (37 ºC, 1 h) and then treated them with 30 μl of hybridization mixture containing 100–300 ng of labelled probes (3 μl), 15 μl 100% formamide, 20×SSC (0.5 μl), 0.1 μl calf thymus DNA, 0.6 μl 50% Dextran and 0.1 μl 10% SDS. We denatured the material at 90 °C for 10 min and carried out hybridization overnight at 37 °C in a humidified chamber. All post-hybridization washes were carried out in 2×SSC, 20% formamide in 0.1×SSC, 0.1×SSC and 4×SSC/0.2% Tween 20, at 42 °C. We detected the probes using a solution of 5% BSA and FITC-conjugated avidin. We then counterstained the chromosomes using 30 µl of 0.2% propidium iodide and mounted the slides in Vectashield antifade (Vector).

## Results

**Figure 3 a–d.**
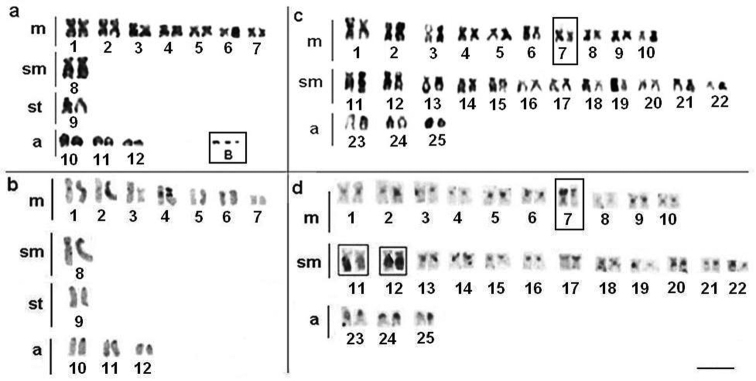
Karyotypes of *Apteronotus* prope *albifrons* and *Rhamphichthys hahni* after: Giemsa-staining **a**, **c** respectively and C-banding **b**, **d**; In evidence, B chromosomes of *Apteronotus* prope *albifrons* **a** and heterochromatic blocks adjacent to the nucleolar regions 4 **b**; **c** terminal secondary constriction on pair 7 and conspicuous heterochromatic regions on pairs 11 and 12 **d** in the karyotype of *Rhamphichthys hahni*. Bar = 5μm.

**Figure 4 a–d.**
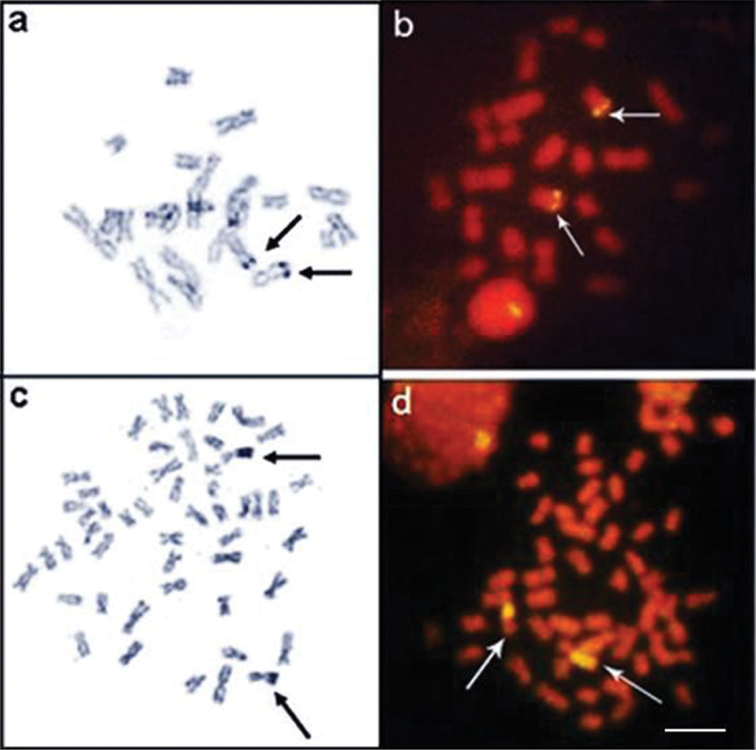
Metaphases showing Ag-NORs-bearing chromosomes and FISH using 18S rDNA probe of: *Apteronotus* prope *albifrons* **a**, **b** and *Rhamphichthys hahni* **c**, **d** Note an evident NOR region-sized heteromorphism of pair 7 of *Rhamphichthys hahni* **c**, **d**. Bar = 5μm.

*Apteronotus* prope *albifrons* showed a diploid number of 24 chromosomes and a karyotype formula of 14m+2sm+2st+6a with a fundamental number (FN) of 42 (Fig. 3a). We observed constitutive heterochromatin distributed in small blocks throughout the pericentromere regions of most of the chromosomes (Fig. 3b) and in conspicuous blocks in the region adjacent to the nucleolar pair 4 secondary constriction (Fig. 3b). The Ag-NOR and 18S rDNA sites were located on the short arm of chromosome pair 4 (Fig. 4a, b), coinciding with the secondary constriction evident in some metaphases.

**Figure 5 a–d.**
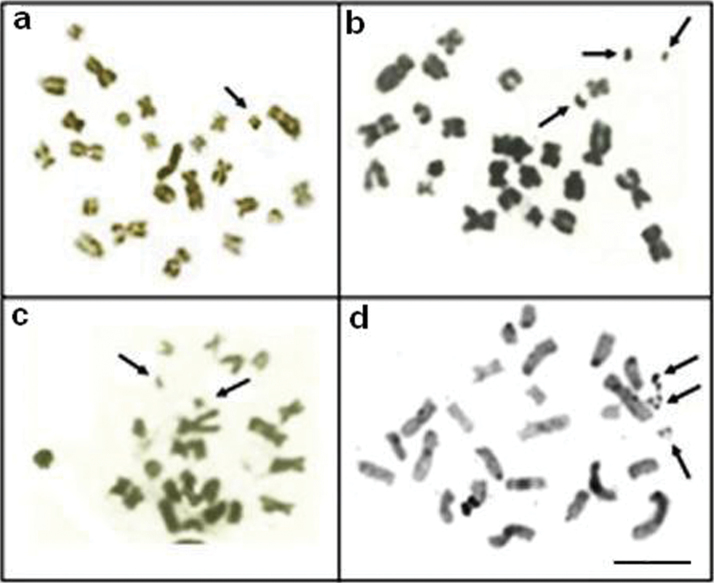
Somaticmetaphases of *Apteronotus* prope *albifrons* stained with Giemsa **a**,**b** and **c** and C-banded **d** showing the B chromosomes (arrows). Bar = 5 μm.

In addition to the normal chromosome complement, we found that three specimens of *Apteronotus* prope *albifrons* had one to three B microchromosomes in their somatic cells, with inter- and intra-individual variations (Fig. 3a, box and Fig. 5a–c). The B chromosomes showed no homology with the other chromosomes of the complement and, morphologically, these chromosomes were classified as acrocentric. We observed constitutive heterochromatin in the pericentromere and terminal position of the extra chromosomes (Fig. 5d).

*Rhamphichthys hahni* showed a diploid number of 50 chromosomes for both sexes and a karyotype formula of 20m+24sm+6a with a fundamental number (FN) of 94 (Fig. 3c). We observed constitutive heterochromatin in the pericentromere regions of most of the chromosomes and conspicuous blocks in three submetacentric chromosomes of the complement and also in the position close to secondary constriction of pair 7 (Fig. 3d). The Ag-NORs and 18S rDNA sites were located in the short arm of chromosome pair 7, coincideing with a terminal secondary constriction (Fig. 3c, box, and Fig. 4 c,d). We also observed an NOR region-sized heteromorphism in this pair (Fig. 4c, d).

**Figure 6 a–c.**
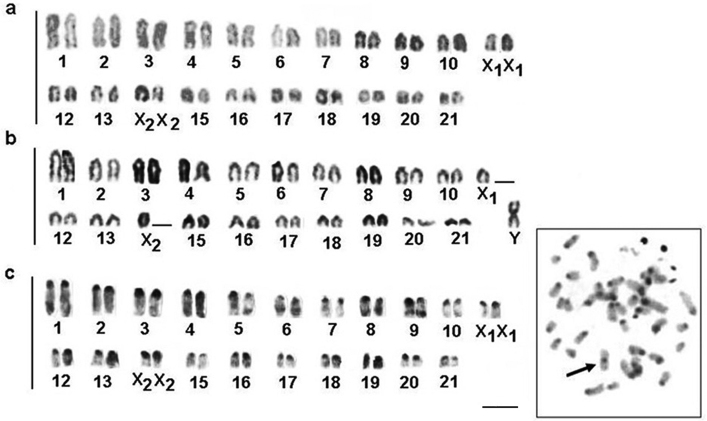
Karyotypes and metaphases of *Brachyhypopomus gauderio* after: Giemsa- staining in female **a** male **b** and C-banding in female **c**. Right, C-banded metaphase showing the Y chromosome (arrow). Bar = 5μm.

*Brachyhypopomus gauderio* presents 2n=42 chromosomes in females and 2n=41 chromosomes in males. The female karyotype showed acrocentric chromosomes only (Fig. 6a) and the male karyotype showed 40 acrocentric chromosomes and one medium-sized metacentric chromosome (Fig. 6b). We found that the fundamental number for both sexes is 42. The difference in karyotype structure between the sexes demonstrates the presence of sex chromosomes, with a metacentric chromosome corresponding to a Y chromosome. Chromosomal pairs 11 and 14 are X_1 _and X_2_ in males and females, respectively. In fact, this condition characterizes a system of multiple sex chromosomes of the type X_1_X_1_X_2_X_2_/X_1_X_2_Y.

**Figure 7 a–d.**
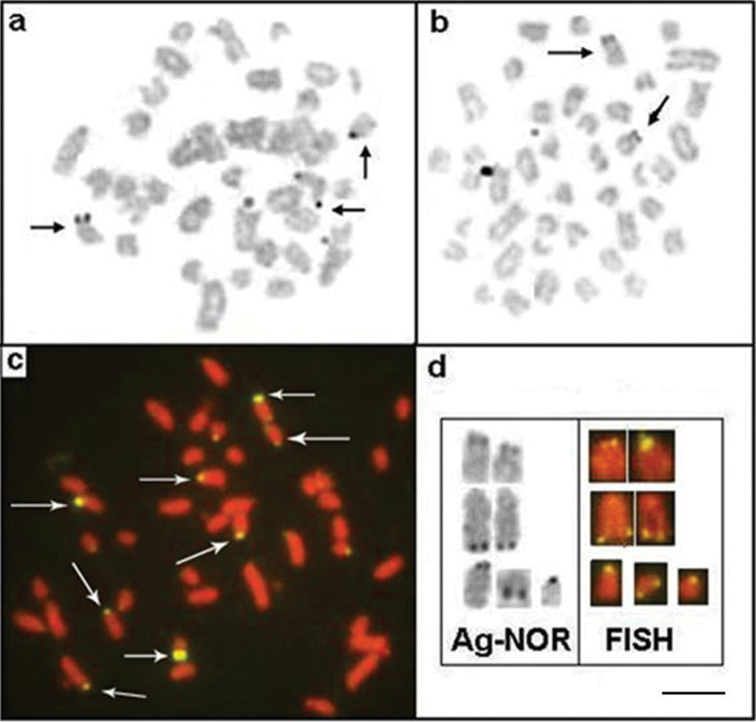
Metaphases of *Brachyhypopomus gauderio* showing NORs regions: Ag-NORs-bearing chromosomes in the male **a** female **b** and eight 18S rDNA sites, arrows **c**; **d** correlation between some Ag-NOR and 18S rDNA regions. Bar = 5μm.

We found C-positive blocks (constitutive heterochromatin) in the pericentromere regions of all chromosomes in males and females (Figure 6c) of *Brachyhypopomus gauderio*, including the pericentromere region of the Y metacentric chromosome (Fig. 6, in the boxes). Furthermore, some chromosomes showed blocks of heterochomatin in the long arm in the interstitial and telomere positions. We observed Ag-NOR sites in the short and long arms of the acrocentric pairs (Fig. 7a, b), and the FISH technique revealed eight fluorescent signals (rDNA sites), including the sites stained by silver (Figure 7c). Figure 6d shows a correlation between some of the Ag-NOR and 18S rDNA sites.

## Discussion

The karyotype structure we describe herein for *Apteronotus* prope *albifrons* (2n=24, 14m+2sm+2st+6a) is similar to the one reported by Howell (1972) from the Amazon basin, but differs from that of a population studied by Almeida-Toledo et al. (1981) from Marajó Island, Pará) (Table 1). These small variations between the chromosome formula described for *Apteronotus albifrons* are expected as a result of different interpretations of the classification of chromosomes when the existence of a secondary constriction in pair 4 is taken into account. Thus, we concluded that the karyotypes of *Apteronotus* prope *albifrons* (in the current study) and *Apteronotus albifrons* (Amazon basin) are similar, except for the presence of B chromosomes in the population inhabited the Parana River. According to Santana (2003), *Apteronotus albifrons* is most likely a complex of closely related cryptic species. If this is applicable to the *Apteronotus* prope *albifrons* populations discussed here, the presence of B chromosomes could be a karyotaxonomic character. The occurrence of B chromosomes in *Apteronotus* prope *albifrons* only could represent a specific characteristic of the population (probably, a geographical variation) in the upper Parana River floodplain.

Our report of B chromosomes in *Apteronotus* prope *albifrons* is the first description of these elements in the Gymnotiformes order. The mitotic instability presented by the B chromosomes of *Apteronotus* prope *albifrons* is likely due to their non-Mendelian behaviour during cell division, which is a common feature attributed to B chromosomes in other species.

The origin of B chromosomes is not yet well understood, although intraspecific origin of B chromosomes in some Neotropical fishes have been suggested as a result of the non-disjunction of an autosome, the formation of isochromosomes, centric fragments resulting from chromosomal rearrangements and amplifications of paracentric regions of a fragmented A chromosome (Camacho 2000, Carvalho et al. 2008).

Heterochromatinization plays an important role in the differentiation of B chromosomes. The B chromosomes are thought to be composed of repetitive sequences (heterochromatin) that lack protein-coding genes. The fact that the B chromosomes of *Apteronotus* prope *albifrons* appeared to be partially heterochromatic does not provide evidence of the presence or absence of coding genes. This would require a detailed molecular cytogenetic analysis with specific probes for FISH. Regarding the pattern of C bands of the other chromosomes of *Apteronotus* prope *albifrons*, we noted a similarity in the location of the heterochromatin when comparing it with the population studied by Almeida-Toledo et al. (1981).

The presence of only one NOR-bearing chromosome pair in *Apteronotus* prope *albifrons* in this study (confirmed by FISH) corresponds to the condition found in an Amazonian basin population of *Apteronotus albifrons* analysed by Almeida-Toledo et al. (1981). The presence of a nucleolar pair in most species of Gymnotiformes is a common pattern (see the review of Arai 2011). We found that the secondary constriction in this pair (no. 4) had a negative C-band pattern. This was also reported by Almeida-Toledo et al. (1981) for this species and for *Electrophorus electricus* Linnaeus, 1766 (Gymnotidae from the Amazon River (Fonteles et al. 2008).

Finally, we analysed the karyotypic data available so far for the Apteronotidae family and observed a relative numerical and structural variability, ranging from 2n=22 and 24 to 52 (Table 1), suggesting an evolutionary history of chromosomal rearrangements. This trend can also be seen when comparing the karyotypic formulas of the *Apteronotus* La Cépede, 1800 and *Parapteronotus* Albert, 2001 genera (Table 1). However, the absence of cytogenetic data for other species of the Apteronotidae family makes it difficult to understand the karyotypic interrelationships and the types of rearrangements that have resulted in the diploid values in this group so far. In addition, cytogenetic information for other species of this family would be helpful for clarifying the origin of the B chromosomes discussed herein.

## The karyotype structure of Rhamphichthys hani and Brachyhypopomus gauderio

Cytogenetic studies in the Rhamphichthyidae family are still scarce and the karyotype of *Rhamphichthys hahni* is reported herein for the first time. The cytogenetic data available for *Rhamphichthysi* Müller et Troschel, 1848show less variation in diploid number and chromosome structure (see Table 1). For example, the comparison of the karyotype of *Rhamphichthys rostratus* Linnaeus, 1766 (Silva 2010) with that of *Rhamphichthys hahni* (2n=50, 20m+24sm+6a) in the current study shows that their karyotype formula are similar. Although these data reflect a relatively more conservative chromosomal evolution in the Rhamphichthyidae family, the diploid numbers of 50–52 chromosomes do not exclude the possibility of rearrangements such as fissions/fusions occurring during the karyotypic evolution of this group, besides those associated with changes in the karyotype formulae.

The presence of NOR in only one pair of chromosomes found in *Rhamphichthys hahni* coincides with the patterns observed in *Rhamphichthys marmoratus* Castelnau, 1855 and *Rhamphichthys rostratus* (Silva 2010). The NOR-sized heteromorphism found in the nucleolar pair (no. 7) in *Rhamphichthys hahni* suggests a structural variation in the number of DNAr cistrons among the homologues. This phenomenon could have originated from an unequal crossing-over, as found in many fish species. For example, among the Gymnotiformes, NOR-sized heteromorphisms were visualized in species of *Eigenmannia* (Foresti et al. 1981, Almeida-Toledo et al. 1996, Foresti 1987) and *Steatogenys* Boulenger, 1898 genera (Cardoso et al. 2011).

The C-band pattern in *Rhamphichthys hahni* is similar to the heterochromatin location in the pericentromere region in many species of Gymnotiformes. The NOR region is associated with heterochromatin, which is frequently found in many fish species. However, three medium-sized submetacentric chromosomes show conspicuous heterochromatic blocks on the long arm. The distribution and amount of heterochromatin may have an important evolutionary role in the chromosomes of many fish species, including sex chromosomes, as reported in *Eigenmannia virescens* (Almeida-Toledo et al. 2001, Silva et al. 2009) and *Steatogenys elegans* Steindachner, 1880 (Cardoso et al. 2011). We found no evidence of sex chromosome differentiation in *Rhamphichthys hahni*, and the heterochromatic blocks on the long arm of the metacentric chromosomes could be useful markers of this species. However, other species have to be analysed.

The chromosome formula discovered by us in *Brachyhypopomus gauderio* corresponds to the data previously reported by Almeida-Toledo et al. (2000b) (see Table 1). There is little cytogenetic information available on the *Brachhypopomus* genus. In addition to the above mentioned species, the karyotype of *Brachhypopomus brevirostris* Steindachner, 1868 from the Amazon basin (Almeida-Toledo 1978) was also recorded karyotype formula; the sex chromosomes have still not been distinguished.

The origin of the multiple sex determination system, X_1_X_1_X_2_X_2_/X_1_X_2_Y in *Brachyhypopomus gauderio* (cited as *Brachyhypopomus pinnicaudatus*) was discussed by Almeida-Toledo et al. (2000b) using C-band, DAPI staining and FISH (with a telomere probe) techniques. Based on these analyses, the authors suggested that the Y chromosome originated from a centric fusion (Robertsonian fusions) involving two average sized acrocentric chromosomes. In the present study, *Brachyhypopomus gauderio* showed a C-band pattern similar to that detected in the population from the Tietê River (Almeida-Toledo et al. 2000b), including the C-positive blocks on the pericentromere region of the Y metacentric chromosome, strengthening the hypothesis discussed above.

The detection of up to eight chromosomes with a fluorescent signal (18S rDNA sites) indicates multiple NOR systems in *Brachyhypopomus gauderio*. Furthermore, the difference in the results between the methodologies (Ag-NOR and FISH) suggests that not all ribosomal DNA sites were active in the previous interphase. Also, chromosomal rearrangements such as translocations and/or transpositions, resulting in the dispersion of ribosomal genes, might explain this variability. No information is provided on the sites of NORs in the *Brachyhypopomus pinnicaudatus* (currently *Brachyhypopomus gauderio*) population analysed by Almeida et al. (2000b); however, in some individuals from an *Eigenmania* sp.1 population Almeida-Toledo et al. (1996) found four chromosomes with NOR regions.
